# InCoB2012 Conference: from biological data to knowledge to technological breakthroughs

**DOI:** 10.1186/1471-2105-13-S17-S1

**Published:** 2012-12-07

**Authors:** Christian Schönbach, Sissades Tongsima, Jonathan Chan, Vladimir Brusic, Tin Wee Tan, Shoba Ranagathan

**Affiliations:** 1 Department of Bioscience and Bioinformatics, Kyushu Institute of Technology, Fukuoka 820-8502, Japan; 2 Biomedical Informatics Research and Development Center, Kyushu Institute of Technology, Fukuoka 820-8502, Japan; 3 National Center for Genetic Engineering and Biotechnology (BIOTEC), National Science and Technology Development Agency (NSTDA), Thailand Science Park, Pathumthani 12120, Thailand; 4 School of Information Technology, King Mongkut's University of Technology Thonburi, Bangkok 10140, Thailand; 5 Cancer Vaccine Center, Dana-Farber Cancer Institute, Boston, MA 02115, USA; 6 Department of Biochemistry, Yong Loo Lin School of Medicine, National University of Singapore, Singapore 117597, Republic of Singapore; 7 Computational Resource Centre (A*CRC), A*STAR, Singapore 138632, Republic of Singapore; 8 Department of Chemistry and Biomolecular Sciences and ARC Centre of Excellence, Macquarie University, Sydney, NSW 2109, Australia

## Abstract

Ten years ago when Asia-Pacific Bioinformatics Network held the first International Conference on Bioinformatics (InCoB) in Bangkok its theme was North-South Networking. At that time InCoB aimed to provide biologists and bioinformatics researchers in the Asia-Pacific region a forum to meet, interact with, and disseminate knowledge about the burgeoning field of bioinformatics. Meanwhile InCoB has evolved into a major regional bioinformatics conference that attracts not only talented and established scientists from the region but increasingly also from East Asia, North America and Europe. Since 2006 InCoB yielded 114 articles in BMC Bioinformatics supplement issues that have been cited nearly 1,000 times to date. In part, these developments reflect the success of bioinformatics education and continuous efforts to integrate and utilize bioinformatics in biotechnology and biosciences in the Asia-Pacific region. A cross-section of research leading from biological data to knowledge and to technological applications, the InCoB2012 theme, is introduced in this editorial. Other highlights included sessions organized by the Pan-Asian Pacific Genome Initiative and a Machine Learning in Immunology competition. InCoB2013 is scheduled for September 18-21, 2013 at Suzhou, China.

## Background

Asia-Pacific Bioinformatics Network (APBioNet) [1] was founded in 1998 during the Pacific Symposium on Biocomputing in Hawaii. Its mission is to promote and advance bioinformatics as a scholarly scientific discipline in the Asia-Pacific region. Since then, APBioNet has been engaged in the development of bioinformatics network infrastructure, primarily through training and organization of conferences. These activities won APBioNet the endorsement of Asia-Pacific Advanced Network (APAN) [2], the acknowledgment of Subcommittee of Biotechnology of Association of Southeast Asian Nations (ASEAN) Committee on Science and Technology (COST) [3] and the regional affiliation with International Society for Computational Biology (ISCB) [4]. Since 2002, the International Conference of Bioinformatics (InCoB) series of conferences has evolved into a major forum for fostering APBioNet's mission and for advancing the theory and practice of bioinformatics. The InCoB community comprises practitioners, scientists and students spanning biology, computation, and biotechnology fields. The progress and maturation of bioinformatics in the Asia-Pacific region are documented in editorials of previous InCoB supplements in *BMC Bioinformatics *[5-10] and *BMC Genomics *Supplements [11-14]. Shortly after InCoB2012, APBioNet was incorporated in Singapore as a public limited liability company. The legal and organizational structure of Asia Pacific Bioinformatics Network Ltd. will ensure quality, sustainability and continuity of its mission to advance bioinformatics across the region and beyond.

### Overview

APBioNet's 11^th ^International Conference on Bioinformatics [14] was held in Bangkok, Thailand on Oct 3-5, 2012. InCoB2012 was co-hosted by Thai government's National Center for Genetic Engineering and Biotechnology (BIOTEC) [15], which is a research center under the National Science and Technology Development Agency (NSTDA) [16] as well as the King Mongkut's University of Technology Thonburi (KMUTT) [17]. The conference was supported by the Thailand Convention & Exhibition Bureau [18], ISCB and the HUGO Pan Asia Population Genomics Initiative (PAPGI) [19]. Highlights of the two PAPGI sessions are included in the editorial of the *BMC Genomics *Supplement [20].

More than 270 delegates attended this year's conference which featured four keynote and five plenary session speakers. Jürg Ott (Rockefeller University USA; Chinese Academy of Sciences, China) presented in his keynote "Statistical approaches to testing and estimating the number of functional variants in complex diseases". Keynote speaker, Yongyuth Yuthavong (BIOTEC; Mahidol University, Thailand) reviewed "Bioinformatics within and beyond biology", tracing the growth of bioinformatics in Thailand. Tatsuhiko Tsunoda (RIKEN Center for Genomic Medicine, Japan) lectured about "Genomic medicine's milestones and future". Sandra L. Baldauf (Uppsala University, Sweden) reviewed her research on "Comparative genomics of microbial eukaryotes". Ram Samudrala (University of Washington, USA) presented in his plenary talk "Computational analysis of novel drug opportunities". David W. Ussery's (Technical University of Denmark) plenary covered the progress and problems of bacterial genome sequence to taxonomy. Juncai Ma (Chinese Academy of Sciences, China) introduced the Global Catalogue of Microorganisms [21] of World Federation for Culture Collections. Yutaka Yasui (University of Alberta, Canada) gave an overview of epistasis exploration as a key element in genome-wide association studies. The plenary talks were concluded by Richard Wintle's (The Hospital for Sick Children, Toronto, Canada) with a presentation about "Old disorders, new approaches: genomic variability in autism spectrum disorder".

Of 123 research paper submissions, 53 were accepted for publication as proceedings articles after peer-review and revision. Forty-eight submissions were rejected and withdrawn. The remaining 22 submissions were resubmitted as posters and accepted along with 49 regular poster submissions. The 25 articles published in this supplement and 28 in *BMC Genomics *Vol. 13 Supplement 7 [22] have been arranged according to the session topics. The best paper title was awarded to Yongjin Li and Jinyan Li for their advancement of disease gene identification using a new random walk model that allows cross-walking between phenotype and gene networks [23]. The runners-up were Chian Y. Teo *et al.*, [24] and Mostafa M. Abbas *et al*. [25], whose works indicated compelling translational potential or theoretical impact, respectively.

#### Proteins, ligands and docking

Vinekar *et al*. applied phylogenetic, homology modelling and molecular dynamics simulation methods to explore the activity regulation across members of the isocitrate dehydrogenase (IDH) family. Their comprehensive analysis provides clues about mechanisms of inactivation of IDH in pathogens such as *M. tuberculosis *[26]. Huang *et al*. contributed the "Prediction and analysis of protein solubility using a novel scoring card method with dipeptide composition" [27]. The new scoring card method outperforms the existing methods in predicting protein solubility and enables the adjustments of the algorithm representing experimental conditions of *in vitro *protein expression.

#### Virtual screening and docking

Similar to previous InCoB conferences, the topic of docking and virtual screening attracted a high number of submissions resulting in six accepted papers. The Malaysian-Scottish collaborative work on the "Discovery of a new class of inhibitors for the protein arginine deiminase type 4 (PAD4) by structure-based virtual screening" [24] was the runner-up for the best paper. The novel overall strategy that combined *in silico *screening with experimental validation of compounds enabled better understanding of treatment options for rheumatoid arthritis. An Egyptian group reported a virtual screening workflow and its application to drug repositioning using the DrugBank [28] data. The authors identified four potential inhibitors of Hepatitis C virus NS5B RNA-dependent RNA polymerase [29]. Lee and Kim generated large-scale reverse docking profiles for 35 well-known ligands used in drugs with currently available yeast and human X-ray crystal protein structures [30]. Their profiles aid the evaluation of binding assays and add value to efforts of applying known drugs to new indications or diseases, a process called drug repositioning. Another useful community resource is the India-based "Sanjeevini" web tool for finding potential lead compounds targeting proteins or DNA [31]. Two papers added the pathway and text mining perspectives to the topic of virtual screening. Nakamura *et al*. developed a novel algorithm for *de novo *predictions of biochemical pathways induced by chemical compounds [32]. The reproducibility of the approach in known pathways raises the potential to discover new reaction pathways. The ChemEx: information extraction system by Supawadee Ingsriswang's group at BIOTEC Thailand bridges virtual screening and docking with image recognition and text mining [33].

#### Motifs, patterns, mutations and bioimaging

An improved method for the exact planted (l, d) motif finding [25] improved the run-time performance. It has implications for next-generation sequence data analysis where hundreds of millions of reads are performed. This submission was a runner-up to the best paper award.

The group of John P. Overington mapped the binding of small molecules to Pfam-A domains of protein targets in the ChEMBL bioactivity database [34]. The evaluation of potential drug targets or high throughput screening results is expected to benefit considerably from this approach.

Two papers reported new approaches to the long-standing problems. Joung *et al*. reported a new algorithm for discovering coherent patterns in gene expression datasets that can be applied to identify potentially related functional groups of genes [35]. Probabilistic latent semantic indexing was used by Su *et al*. to predict the nuclear/non-nuclear localization potential of proteins [36].

A study of muscle remodeling in *Drosophila *metamorphosis using bioimage informatics by Martin Wasser's group [37] introduced a workflow that can handle spatial and temporal dimensions of cellular dynamics to support the quantitative analysis phenotypic changes.

#### Pathways and networks

The temporal dimension of protein-protein interactions (PPI) is an important but less-studied topic within the PPI network analysis. Koh *et al*. classified hubs depending on their temporal participation in complexes and reported associations between constitutive protein expressions and their temporal reusability in yeast [38]. A related case study of yeast cell cycle dynamics demonstrates a new classification of proteins using their temporal participation in complexes [39]. CMPF (Class-switching Minimized Pathfinder) is based on a path weighting method that elegantly addresses the problem of sequences of enzymes being present or absent in a given pathway, while taking into account their localization and the species [40].

#### Disease informatics

Contributions ranged from mathematical models of immune responses to a diagnostic prototype system. Kaewkamnerd *et al*. [41] introduced a device for detection and classification of malaria parasite species from blood film. The prototype version can assist clinicians in diagnosing malaria. Lai and co-workers developed and implemented a deterministic algorithm that solves problem of zero-recombinant haplotype configuration for a general pedigree in linear time [42]. Prediction of discontinuous B-cell epitopes is a long-standing problem. "BeTop" model draws on statistical ideas, graph clustering algorithms and supervised learning approaches to predict conformational B-cell epitopes using planarity information [43]. An Italian group with a track record in immune system modelling proposed a new nonlinear ordinary differential equation model that simulates the competition between the immune system and mammary carcinoma under different therapeutic vaccination conditions [44]. The model has potential use in optimizing vaccination courses and minimizing side effects.

#### Transcriptomics, genome-wide association studies, miRNAs and small RNAs

Predictably, most contributions covering these three topics were submitted to the *BMC Genomics *track. One web-based EST annotation system was deemed to be of interest for general bioinformatics community and has been published in this issue. The TranSeqAnnotator [45] can cope with large-scale next-generation sequencing EST data sets and has been applied to the functional annotation and analysis of parasitic helminth ESTs.

#### Emerging bioinformatics technologies and applications

This topic includes a variety of works that demonstrate the utility of technology or software for solving biological problems or that support bioinformatics education. El-Kalioby *et al*. show-cased a cloud computing application called elasticHPC. The package comes with applications and libraries that can be used in training (e.g. parallel programming), data processing or building clusters in the cloud [46]. A fast multidimensional scaling for genome-scale data system by Park *et al*. [47] demonstrated divide-and-conquer, random and MaxMin sampling options to reduce the dimensions of large-scale genomic data. A Malaysian group reported the results of a freshwater algae classification feasibility study at a lake near Kuala Lumpur that utilized an artificial neural network approach in image processing of algae sizes and shapes [48]. A phylogenetic reconstruction by Biswal *et al*. [49] represents a case study of a successful molecular morphometrics application concerning the taxonomy of *Hydatellaceae*, a family of small aquatic flowering plants.

### Immunoinformatics and machine learning in immunology competition

In step with the growing utilization of immunoinformatics in immunology and vaccine research InCoB allocated since 2010 a dedicated session. This year, the session Immunological Methods included two oral presentations on novel methods to predict major histocompatibility complex (MHC) class I binding peptides, one talk introducing an analysis of VDJ rearrangements to infer phased haplotypes and an overview of results obtained from the 2^nd ^Machine Learning Competition in Immunology 2012. The competition [50] was organized by Vladimir Brusic and co-sponsored by InCoB2012. The details and data sets are available at the Dana-Farber Repository for Machine Learning in Immunology site [51]. Participants were asked to predict peptides naturally processed by MHC class I pathway for eight target MHC molecules. The NetMHC 3.2 server (1D-BENCH) results were used as a benchmark method [52]. Of 32 prediction methods six were determined as winners that will be published along a special issue of the *Journal of Immunological Methods *in 2013. The papers that were orally presented in InCoB2012 will be published in the *Journal of Immunological Methods *as regular papers to disseminate computational methods amongst the immunology community.

### Forum discussion on bioinformatics resources for teaching, training and research

The APBioNet mission of advancing bioinformatics as a scholarly scientific discipline includes, besides conference and workshop organization, the development of a bioinformatics network infrastructure and training programs. The forum discussion format provided the opportunity to introduce the audience to APBioNet resources and activities. The available resources include BioSLAX bioinformatics tools on Linux live OS [7,53], CanalAVIST system to deliver lectures and streaming videos within the framework of ASEAN Virtual Institute for Science and Technology (AVIST) [54], WizFolio bibliographic management and research collaboration tool [55] and various grid applications of bioinformatic tools. APBioNet actively supports standards and standardization, key issues in bioinformatics, with its Minimum Information about a Bioinformatics Investigation [56], AuthorID [57] and DocID [58] registry, and BioDB100 initiative of 100 MIABI-compliant integrated databases [59]. On the educational front APBioNet engages regularly in workshops ranging from introductory bioinformatics to advanced topics such as BioConductor and parallel computing in R language. The Overseas Research Advancement Programme and Travel Grant (ORAP) supports the travel cost of several graduate students and post-doctoral researchers affiliated with institutions of the Asia-Pacfic region enabling broader attendance of InCoB or other conferences endorsed by APBioNet, and train abroad programs. In support of a global strategic organization for bioinformatics training, APBioNet recently signed along 18 other bioinformatics or computational biology-related networks and societies a memorandum of understanding that led to the establishment of Global Organisation for Bioinformatics Learning, Education & Training (GOBLET) [60]. GOBLET aims to build a truly global, sustainable support structure for bioinformatics educators/trainers and students/trainees in addition to acting as hub for fund gathering. These resources and activities ensure that APBioNet is well positioned to support and represent the Asia-Pacific region in the global research, resource sharing or barrier-free resource access, education, training, and public awareness in the bioinformatics field.

### Future conferences

Bids to host future InCoB conferences as a single event or in conjunction with another conference/workshop are always welcome. Documents for tendering a bid are available on APBioNet's website [1]. Of two excellent proposals for hosting InCoB2013 in Suzhou or Singapore, the InCoB Bid Screening Committee recommended the one from Suzhou with the theme "Biomedical Informatics in the Big Data Era: Data-Driven Biology and Medicine". Calls for participation and manuscript submissions for InCoB2013 scheduled to be held September 18-21, 2013 in Suzhou, about 100 km West of Shanghai, will be posted on the APBioNet website [1].

Additional file 1.

## Competing interests

The authors were organizers, co-chairs, and/or session chairs of InCoB2012. TWT is a founding Director of Asia Pacific Bioinformatics Network, Ltd. All authors declare they have no other conflict of interest.

## Authors' contributions

CS, VB and SR wrote this editorial. CS, SR, ST, JC and TWT served as co-editors for the InCoB2012 supplement issues with SR as the lead editor. CS and SR managed the manuscript submission, peer-review and editorial decision processes as superchairs of EasyChair Conference System. VB organized the machine learning competition in immunology. All authors have seen and approved the manuscript.

## Supplementary Material

Additional file 1 Click here for file
