# Supplementary material for: InCoB2012 Conference: from biological data to knowledge to technological breakthroughs
Source: BMC Bioinformatics. 2012 Dec 7;13(Suppl 17):S1. doi: 10.1186/1471-2105-13-S17-S1 (PMC3521245; doi:10.1186/1471-2105-13-S17-S1)
Supplement: Additional file 1 [file 1471-2105-13-S17-S1-S1.pdf]

Additional file 1.

We thank all members of InCoB2012 Scientific Program Committee and 34 expert referees for their enthusiasm and constructive criticism of manuscripts submitted to be considered for publication in the InCoB2012 supplement issues of BMC Bioinformatics and BMC Genomics.

<u>InCoB2012 Scientific Program Committee members</u>: Shandar Ahmad (National Institute of Biomedical Innovation, Japan), Tatsuya Akutsu (Kyoto University, Japan), Shunsuke Aoki (Kyushu Institute of Technology, Japan), Nicola Armstrong (Garvan Institute, Australia), Vladimir Bajic (King Abddulah University of Science and Technology, Saudi Arabia), Christopher Baker (University of New Brunswick, Canada), Sergio Baranzini (University of California San Francisco, USA), Alex Bateman (Wellcome Trust Sanger Institute, UK), Michael A Beer (Johns Hopkins University, USA), Jong Bhak (Theragen BiO institute, R.O. Korea), Vladimir Brusic (Dana-Farber Cancer Institute, USA), Zhi-Wei Cao (Shanghai Center for Bioinformatics Information Technology, P.R. China), Filippo Castiglione (Institute for Computing Application, CNR, Italy), Jonathan Chan (King Mongkut's University of Technology Thonburi, Thailand), Wasun Chantratita (Mahidol University, Thailand), Alan Christoffels (South African National Bioinformatics Institute, South Africa), Pawan Dhar (Symbiosis International University, India), Frank Eisenhaber (Bioinformatics Institute, A*STAR, Singapore), Mahmoud Elhefnawi (National Research Centre Cairo, Egypt), Mohd Firdaus-Raih (Universiti Kebangsaan, Malaysia), Andrew French (University of Nottingham, UK), Ge Gao (Peking University, P.R. China), Pascale Gaudet (Swiss Institute of Bioinformatics, Switzerland), Susumu Goto (Kyoto University, Japan), M Michael Gromiha (Indian Institute of Technology Madras, India), Marsia Gustiananda (Eijkman Institute for Molecular Biology, Indonesia), Timothy Hancock (Kyoto University, Japan), Yongqun He (University of Michigan, USA), Chia-Lang Hsu (National Yang Ming University, Taiwan), Wenlian Hsu (Academia Sinica, Taiwan), Chun-Hsi Huang (University of Connecticut, USA), Ming-Jing Hwang (Academia Sinica, Taiwan), Asif M Khan (Perdana University, Malaysia), Javed Khan (Macquarie University, Australia), Tsung-Fei Khang (University of Malaya, Malaysia), Daisuke Kiga (Tokyo Institute of Technology, Japan), Akira Kinjo (Osaka University, Japan), Kengo Kinoshita

(Tohoku University, Japan), Tetsuya Kobayashi (University of Tokyo, Japan), Akihiko Konagaya (Tokyo Institute of Technology, Japan), Shinji Kondo (RIKEN Omics Center, Japan), Anton Kratz (RIKEN Omics Center, Japan), Gaurav Kumar (Macquarie University, Australia), Hiroyuki Kurata (Kyushu Institute of Technology, Japan), Igor Kurochkin (Bioinformatics Institute, A*STAR, Singapore), Chee Keong Kwoh (Nanyang Technological University, Singapore), Chih Lee (University of Connecticut, USA), Jinyan Li (University of Technology, Sydney), Xiaoli Li (Institute for Infocomm Research), Chidchanok Lursinsap (Chulalongkorn University, Thailand), Sorayya Malek (University of Malaya, Malaysia), Hiroshi Mamitsuka (Kyoto University), Hideo Matsuda (Osaka University, Japan), Asawin Meechai (King Mongkut's University of Technology Thonburi, Thailand), Bui Q Minh (Center for Integrative Bioinformatics Vienna, Austria), Lenny Moise (University of Rhode Island, USA), Santo Motta (University of Catania, Italy), Hampapathalu Nagarajaram (Centre for DNA Fingerprinting and Diagnostics, India), Kenta Nakai (University of Tokyo, Japan), Haruki Nakamura (Osaka University, Japan), Sheila Nathan (Universiti Kebangsaan, Malaysia), Yasushi Okazaki (Saitama Medical University, Japan), Prasit Palittapongarnpim (Mahidol University, Thailand), Francesco Pappalardo (University of Catania, Italy), Ashwini Patil (University of Tokyo, Japan), Konstantin Pervushin (Nanyang Technological University, Singapore), Nikolai Petrovsky (Flinders Medical Centre, Australia), Vongsakorn Poonpiriya (National Science and Technology Development Agency, Thailand), Gajendra PS Raghava (Institute of Microbial Technology, India), Shoba Ranganathan (Macquarie University, Australia), Wickneswari Ratnam (University Kebangsaan, Malaysia), Timothy Ravasi (King Abdullah University of Science and Technology, Saudi Arabia), Yasubumi Sakakibara (Keio University, Japan), Meena Sakharkar (Tsukuba University, Japan), Susanna-Assunta Sansone (University of Oxford, UK), Daniele Santoni (Institute for System Analysis and Computer Science "Antonio Ruberti", CNR, Italy), Santoni (Institute for System Analysis and Computer Science "Antonio Ruberti, CNR, Italy), Christian Schönbach (Kyushu Institute of Technology, Japan), Masakazu Sekijima (Tokyo Institute of Technology, Japan), Shahir Shamsir (University Teknologi Malaysia, Malaysia), Tetsuo Shibuya (University of Tokyo, Japan), Kazuyuki Shimizu (Kyushu Institute of Technology, Japan), Ramanathan Sowdhamini (National Centre for Biological Sciences, India), Narayanaswamy Srinivasan (Indian Institute of Science, India), Daron M Standley (Osaka University, Japan), Durai Sundar (Indian

Institute of Technology Delhi, India), Y-H Taguchi (Chuo University, Japan), Takako Takai-Igarashi (Tokyo Medical and Dental University, Japan), Yoichi Takenaka (Osaka University, Japan), Tin Wee Tan (National University of Singapore, Singapore), Visit Thongboonkerd (Mahidol University, Thailand), Paolo Tieri (University of Bologna, Italy), Joo Chuan Tong (Institute for Infocomm Research, A*STAR, Singapore), Sissades Tongsima (National Center for Genetic Engineering and Biotechnology, Thailand), Ikuo Uchiyama (National Institute for Basic Biology, Japan), Pascal Vallotton (Commonwealth Scientific and Industrial Research Organisation , Australia), Chandra Verma (Bioinformatics Institute, A*STAR, Singapore), Lawrence Wee (National University of Singapore, Singapore), Ratnam Wickneswari (University Kebangsaan, Malaysia), Li Xiaoli (Institute for Infocomm Research, A*STAR, Singapore), Chao Xie (National University of Singapore), Ueng-Cheng Yang (National Yang Ming University, Taiwan), Kei Yura (Ochanomizu University, Japan), Guang Lan Zhang (Dana-Farber Cancer Institute, USA) and Shanfeng Zhu (Fudan University, P.R. China).

<u>Additional expert referees</u>: Arsen Batagov (Bionformatics Institute, A*STAR, Singapore), Jake Bundy (Imperial College London, UK), Stephen Carr (Research Complex at Harwell, UK), Brad Chapman (Harvard Univeristy, USA), Caster Chen (Academia Sinica, Taiwan), Li Chen (National Institutes of Health, USA), Hirokazu Chiba (National Institute for Basic Biology, Japan), Surasak Chunsrivirot (National Center for Genetic Engineering and Biotechnology, Thailand), Alessandro Cincotti (Advanced Institute of Science and Technology, Japan), Worrawat Engchuan (King Mongkut's University of Technology Thonburi, Thailand), Darren Flower (Aston University, UK), Akira Funahashi (Keio University, Japan), Ting Gong (Virginia Tech, USA), Hsuan-Cheng Huang (National Yang-Ming University, Taiwan), Boris Jankovic (King Abdullah University of Science and Technology, Saudi Arabia), Adwait Joshi (National Centre for Biological Sciences, India), Agnes Kirchhoff (Botanic Garden & Botanical Museum Berlin-Dahlem, Germany), Artjom Klein (University of New Brunswick, Canada), Ke-Shiuan Lynn (Academia Sinica, Taiwan), Koji Oda (Taisho Pharmaceutical Co., Ltd.), Lars R Olsen (Dana-Farber Cancer Institute, USA), Seong S Ong (Universiti Kebangsaan, Malaysia), Marzio Pennisi (University of Catania, Italy), Jittima Piriyapongsa (National Center for Genetic Engineering and

Biotechnology, Thailand), Niko Popitsch (Center of Integrative Bioinformatics Vienna, Austria), Alexandre Riazanov (University of New Brunswick, Canada), Sebastian Schmeier (King Abdullah University of Science and Technology, Saudi Arabia), Torsten Seemann (Monash University, Australia), Vorasuk Shotelersuk (Chulalongkorn University, Thailand), Zhiqun Tang (Bionformatics Institute, A*STAR, Singapore), Alberto Termanini (European Institute of Oncology, Italy), Dario Vianello (University of Bologna, Italy), Jianmin Wu (Garvan Institute, Australia) and Ming Zhang (University of Georgia, USA).
